# Policosanol Stimulates Osteoblast Differentiation via Adenosine Monophosphate-Activated Protein Kinase-Mediated Expression of Insulin-Induced Genes 1 and 2

**DOI:** 10.3390/cells12141863

**Published:** 2023-07-15

**Authors:** Kyeong-Min Kim, Young-Ju Lim, Won-Gu Jang

**Affiliations:** 1 Department of Biotechnology, School of Engineering, Daegu University, Gyeongsan 38453, Republic of Korea; kkm97655@naver.com (K.-M.K.); limyj1179@hanmail.net (Y.-J.L.); 2 Research Institute of Anti-Aging, Daegu University, Gyeongsan 38453, Republic of Korea

**Keywords:** policosanol, osteoblast differentiation, AMPK, INSIG, zebrafish

## Abstract

Policosanol is known as a hypocholesterolemic compound and is derived from plants such as sugar cane and corn. Policosanol can lower blood pressure or inhibit adipogenesis, but its effect on osteogenic differentiation and the molecular mechanism is unclear. This study aims to investigate the effect of policosanol on osteogenic differentiation in MC3T3-E1 cells and zebrafish models. Administration of policosanol into MC3T3-E1 induced the expression of the osteogenic genes such as distal-less homeobox 5 (Dlx5) and runt-related transcription factor 2 (Runx2). Alkaline phosphatase activity and extracellular mineralization also increased. Policosanol promoted activation of adenosine monophosphate-activated protein kinase (AMPK) and insulin-induced genes (INSIGs) expression and regulation of INSIGs modulated osteoblast differentiation. AMPK activation through transfection of the constitutively active form of AMPK (CA-AMPK) increased INSIGs expression, whereas policosanol-induced INSIGs expression was suppressed by inhibitor of AMPK (Com. C). Furthermore, the osteogenic effects of policosanol were verified in zebrafish. Amputated caudal fin rays were regenerated by policosanol treatment. Taken together, these results show that policosanol increases osteogenic differentiation and contributes to fin regeneration in zebrafish via AMPK-mediated INSIGs expression, suggesting that policosanol has potential as an osteogenic agent.

## 1. Introduction

Bone is a dynamic structure that is constantly being remodeled by the interaction between bone formation by osteoblasts and bone resorption by osteoclasts over a lifetime [1,2]. Osteoblasts differentiate from mesenchymal stem cells into osteocytes [3,4]. Osteoblast differentiation proceeds through three stages: proliferation, matrix maturation, and mineralization [5]. This process involves several transcription factors and cytokines, including DNA-binding protein inhibitor (Id1), distal-less homeobox 5 (Dlx5), runt-related transcription factor 2 (Runx2), and bone morphogenetic proteins (BMPs) [6,7]. In addition, there are reports that plant-derived natural products or synthetic drugs positively affect osteoblast differentiation [8,9,10].

Policosanol is a very long saturated fatty alcohol found extensively in many plants, such as sugarcane, corn, and rice bran. Policosanol consists of 24 to 34 carbon atoms, and it is named triacontanol, hexacosanol, heptacosanol, octacosanol, or policosanol according to the number of carbons contained in the compound [11,12]. The major function of policosanol is to increase high-density lipoprotein (HDL) cholesterol and, acting as a hypocholesterolemic agent, to lower low-density lipoprotein (LDL) cholesterol [13,14]. In addition, it has been reported that policosanol improves cardiovascular diseases such as atherosclerosis, lowers blood pressure, and inhibits hepatic lipid accumulation [14,15,16,17]. In studies of bone, there is a report that it prevents bone loss in ovariectomized rats [18]. However, how policosanol affects bone formation is unclear.

The adenosine monophosphate-activated protein kinase (AMPK) is a protein that acts as a representative energy sensor in cells. It is activated according to [AMP]/[ATP] ratio and regulates biological responses in various cells [19,20]. Metabolically, AMPK also plays a variety of roles. In glucose metabolism, it functions as a glucose sensor for insulin secretion in pancreatic β cells. In lipid metabolism, AMPK is known to promote lipolysis by inactivating acetyl-CoA carboxylase1 (ACC1) [21,22]. Furthermore, in bone research, AMPK has been reported as a factor that increases osteoblast differentiation [8,23]. In particular, activation of AMPK induces protein stabilization through phosphorylation of Runx2 or phosphorylation of Smad1/5/8 in the BMP signaling pathway, thereby increasing osteoblast differentiation [23,24].

Insulin-induced genes (INSIGs) included in the endoplasmic reticulum (ER) are known to have two subtypes, INSIG1 and 2, which are structurally almost similar. [25]. It has been reported that INSIGs inhibit β-hydroxy β-methylglutaryl (HMG)-CoA reductase in cholesterol metabolism. They act by inhibition of sterol regulatory element-binding protein (SREBP) and SREBP-cleavage activating protein (SCAP) [26,27,28]. Moreover, these INSIGs form a complex with SCAP to reduce the expression of lipogenic genes, which inhibits adipocyte differentiation [29,30]. In β cells, INSIG also showed a protective effect against glucolipotoxicity by regulating the expression of SREBP [31]. However, it has not been reported what role INSIGs play in osteoblast differentiation.

According to our previous findings, policosanol inhibits inorganic phosphate (PI)-induced vascular smooth muscle cell (VSMC) calcification [32]. However, it is not known what effect policosanol has on osteoblast differentiation. Therefore, the aim of this study was to investigate the effect of policosanol on osteoblast differentiation and its molecular mechanism. In this study, we investigated the effect of policosanol on osteoblast differentiation by determining the osteogenic genes expression after policosanol treatment. We demonstrated that policosanol promotes osteogenic differentiation via AMPK-mediated INSIGs expression.

## 2. Materials and Methods

### 2.1. Materials

The policosanol was purchased from BOC Sciences (Shirley, NY, USA). Minimum Essential Medium Eagle with Alpha modification (αMEM), phosphate-buffered saline (PBS), and 0.25% trypsin- EDTA were purchased from Gibco-BRL (Grand Island, NY, USA). AmpiGene^TM^ qPCR Green Mix Hi-ROX was purchased from Enzo (Farmingdale, NY, USA). Runx2 and β-actin antibodies were acquired from Santa Cruz Biotechnology (Santa Cruz, CA, USA). Antibody against Dlx5 was purchased from Cell Signaling Technology (Cambridge, MA, USA), and INSIG1 and INSIG2 antibodies were purchased from Abcam (Cambridge, MA, USA). Compound C (Com. C), an AMPK inhibitor, was purchased from Abcam.

### 2.2. Cell Culture

The mouse-calvaria-derived preosteoblast cell line MC3T3-E1 cells (ATCC, Manassas, VA, USA) were cultured with αMEM supplemented with 10% fetal bovine serum (FBS), 100 units/mL penicillin, and 100 μg/mL streptomycin (Gibco-BRL, Grand Island, NY, USA) in humidified air containing 5% CO_2_ at 37 °C. For osteoblast differentiation, ascorbic acid (AA; 50 μg/mL, Sigma Aldrich, St. Louis, MO, USA) and β-glycerophosphate (β-GP; 5 mM, Sigma Aldrich) were added to the culture medium. The medium was changed every 2 or 3 days. To determine the effects of policosanol, cells were cultured in medium containing 50 μg/mL policosanol. The medium with policosanol was replaced every 2 days.

### 2.3. Real-Time RT-PCR (qPCR) Analysis

Total RNA was isolated from cultured cells using TRIzol reagent (Bio Science Technology, Daegu, Korea) and isolation was performed according to the manufacturer’s instructions. The reverse-transcription reaction was performed using 3 μg of total RNA. Real-time RT-PCR (qPCR) was performed using TOPreal™ SYBR Green qPCR PreMIX (Enzynomix, Daejeon, Korea) on the LightCycler Nano Instrument (Roche, Mannheim, Germany). The primer sequences of qPCR were as follows: *β-actin* forward, 5′-TTC TAC AAT GAG CTG CGT GTG-3′ and reverse, 5′-GGG GTG TTG AAG GTC TCA AA-3′, *Dlx5* forward, 5′-GCC CAC CAA CCA GCC AGA GA-3′ and reverse, 5′-GCG AGG TAC TGA GTC TTC TGA AAC C-3′, *Runx2* forward, 5′-AGA TGA CAT CCC CAT CCA TC-3′ and reverse, 5′-GTG AGG GAT GAA ATG CTG G-3′, *INSIG1* forward, 5′-CCT TGA CTT TAG CAG CCC TCT-3′ and reverse, 5′-TCG TCC TAT GTT TCC CAC TGT-3′, *INSIG2* forward, 5′-GGA GTC ACC TCG GCC TAA AAA-3′ and reverse, 5′-AGT TCA ACA CTA ATG CCA GGA-3′. The mRNA expression was normalized to that of β-actin, and data were analyzed using the ^ΔΔ^C_T_ method.

### 2.4. Transient Transfection and the Promoter Assay

Overexpression vectors (pCMV-INSIG1 and 2, PCDNA3.0 CA-AMPK) were purchased from the Korea Human Gene Bank (KRIBB, Daejeon, Korea). Each of the two types of INSIG siRNA (siINSIG1-1, 2, siINSIG2-1, 2) was obtained from Bioneer (Daejeon, Korea). Cultured cells were transiently transfected with the respective overexpressing or reporter plasmids using Lipofectamine 2000 (Invitrogen, Carlsbad, CA, USA) [33] and the cells were harvested 42 h after transfection. Promoter assay using luciferase as a measure of promoter activity was measured using a Dual-Luciferase^®^ Reporter Assay System (Promega, Madison, WI, USA) and a luminometer following the manufacturer’s instructions.

### 2.5. Alkaline Phosphatase (ALP) Staining

To determine ALP activity, MC3T3-E1 cells were cultured at a concentration of 5 × 10^4^ per well of a 24-well cell culture plate. Thereafter, 50 μg/mL AA, 5 mM β-GP and/or 50 μg/mL policosanol were treated according to conditions and cultured for 8 days. Cultured cells were fixed using 4% formaldehyde for 5 min at room temperature (RT), and rinsed twice with distilled water, followed by alkaline phosphatase (ALP) chromogen BCIP^®^/NBT solution (Sigma Aldrich) was added for 30 min in a light-blocking state. After washing, the area of stained cells was measured using an Epson Perfection V37 scanner (Seiko Epson, Suwa, Japan). The stained area was graphed after quantification of the stained area using the tracing tool of ImageJ 1.50i software.

### 2.6. Alizarin Red s Staining for Mineralization In Vitro

To measure mineralization, MC3T3-E1 cells were cultured at a concentration of 5 × 10^4^ per well of a 24-well cell culture plate. After cell seeding, 50 μg/mL AA, 5 mM β-GP, and/or 50 μg/mL policosanol were treated according to conditions and cultured for 21 days. Cells were then fixed using 4% formaldehyde for 5 min at RT. After fixation, cells were rinsed three times with triple-distilled water and stained using 2% alizarin red s (ARS) solution (Sigma Aldrich) for 30 min at RT. After additional washing, the stained cells were imaged using an Epson Perfection V37 scanner (Seiko Epson, Suwa, Japan). The stained area was graphed after quantification of the stained area using the tracing tool of ImageJ 1.50i software.

### 2.7. Western Blotting

Total cell extracts were harvested using an EzRIPA Lysis kit (ATTO Technology, Tokyo, Japan) and then centrifuged at 15,000× *g* for 10 min at 4 °C. Total proteins were quantified using the Bradford assay, separated by 10~12% sodium dodecyl sulfate-polyacrylamide gel electrophoresis (SDS-PAGE), and transferred to polyvinylidene fluoride (PVDF) membranes. After blocking using 5% skimmed milk in Tris-buffered saline containing Tween 20 (TBST), the membranes were incubated with specific primary antibodies at a dilution of 1:1000, followed by secondary antibodies at a dilution of 1:2500. Signals were detected using enhanced chemiluminescence (ECL) reagents (Advansta, Menlo Park, CA, USA). Densitometric analysis of the specific protein bands was carried out using a FUSION Solo analyzer system (Vilber Lourmat, Eberhardzell, Germany).

### 2.8. Fish Maintenance

Wild-type zebrafish (Danio rerio) of 3.4~4.4 cm total length (considered to be adult) were maintained with a photoperiod of 14 h light/10 h dark. For measuring the bone regeneration, adult zebrafish were incubated at three fish per tank in 300 mL at 30 ± 1 °C. During the experiments, zebrafish were fed daily with Artemia nauplii (Artemia salina) and Tetra Bits complete. Three-month-old zebrafish were fixed in iced cold water for 3~5 s, and half of the total caudal fin rays (lepidotrichia) were amputated, after which the fish were fed daily with Tetra Bits complete, and the tank water was changed every 2 days. All fishes were maintained following the institutional guidelines of the Committee for Laboratory Animal Care and Use of Daegu University (DUIACC-12020/4-0313-006).

### 2.9. ARS Staining in Zebrafish

ARS staining was used to investigate whether bone regeneration was induced by policosanol. Policosanol (50 μg/mL or 100 μg/mL) or dimethyl sulfoxide (DMSO) at a final concentration of 1% was added to the fish tank (300 mL at 30 ± 1 °C; three fish per tank) daily for 6 days. Fish were removed from the tank and incubated in 4% neutral buffered formaldehyde at 4 °C for 24 h to fix the specimens. To visualize mineralization, fixed zebrafish were macerated with 1% potassium hydroxide and 3% hydrogen peroxide for 12 h, after which specimens were treated with 25% saturated sodium tetraborate for 2 h. After washing, all specimens were stained for 15–25 min with 1% potassium hydroxide and 1 mg/mL ARS and then were rinsed three times in water [23]. Regenerated zebrafish caudal fin was quantified by measuring the length of the stained area based on the cut surface.

### 2.10. Statistical Analysis

All experiments were performed in triplicate. Data were analyzed using Student’s *t*-test or analysis of variance analyses followed by Duncan’s multiple comparison tests. A *p* value < 0.05 was regarded as significant. Results are expressed as the mean ± SD.

## 3. Results

### 3.1. Policosanol Increases Osteoblast Differentiation in MC3T3-E1 Cells

**Figure 1:**
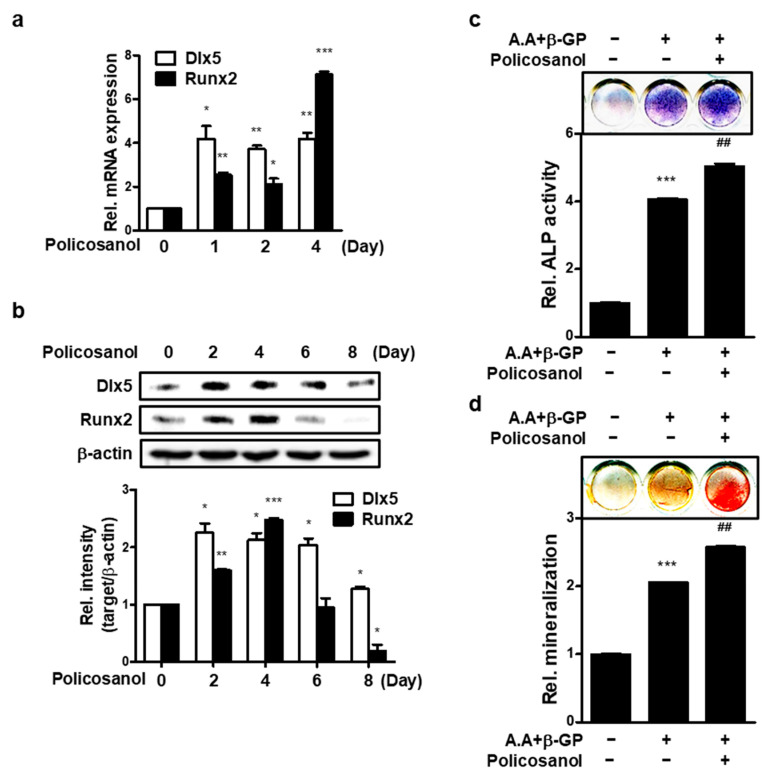
Policosanol stimulates osteoblast differentiation. (**a**) MC3T3-E1 cells were treated with 50 μg/mL of policosanol for 4 days and the expression of mRNA encoding *Dlx5* and *Runx2* was measured using qPCR. (**b**) Western blotting was performed after treatment of MC3T3-E1 cells with 50 μg/mL for 8 days. (**c**) ALP staining was performed after treatment of cells with ascorbic acid (A.A), β-glycerophosphate (β-GP), and 50 μg/mL policosanol for 8 days. (**d**) Extracellular mineralization was determined using ARS staining. Cells were treated with A.A, β-GP, and 50 μg/mL policosanol for 21 days. * *p* < 0.05, ** *p* < 0.01, *** *p* < 0.001 compared with the untreated control, ^##^ *p* < 0.01 compared with the A.A and β-GP-treated group. Data represent the mean ± SD of three individual experiments.

To investigate osteogenic effect of policosanol in MC3T3-E1 cells, osteogenic markers by treatment of policosanol were measured. The expression of osteogenic genes, such as *Dlx5* and *Runx2*, was measured by qPCR analysis. As shown in Figure 1a, policosanol induced the mRNA expression of osteogenic markers such as *Dlx5* and *Runx2*. Policosanol treatment showed a four-fold increase in mRNA expression of *Dlx5* for 1~4 days. The expression of *Runx2* increased about two-fold on the first and second days and showed the highest expression of about seven-fold on the fourth day. At the protein level, policosanol treatment showed a 2-fold increase in Dlx5 expression by day 6, and an increase of about 1.2-fold by day 8. Runx2 showed an increase until day 4 and then decreased thereafter (Figure 1b). As shown in Figure 1c, ALP activity was increased four-fold by A.A+β-GP treatment, which is an osteoblast differentiation condition, and further increased by five-fold when policosanol was additionally treated. Mineralization showed a 2-fold increase in A.A+β-GP treatment, and a 2.5-fold increase in policosanol treatment (Figure 1d).

### 3.2. Policosanol Induces Osteoblast Differentiation via AMPK Phosphorylation

**Figure 2:**
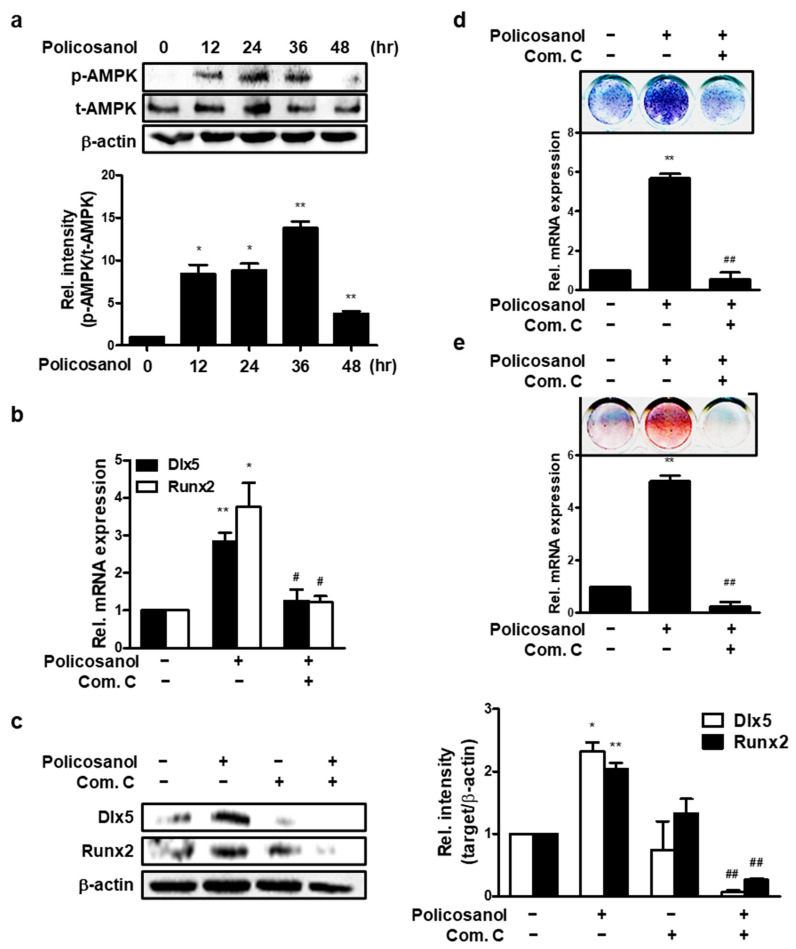
Policosanol increases osteoblast differentiation through AMPK activation. MC3T3-E1 cells were treated with 50 μg/mL policosanol and/or 1 μM Com. C. (**a**) Cells were harvested to determine the protein levels. Western blotting assay was performed using the indicated antibodies. (**b**) Policosanol- and Com-C-treated cells were cultured for 4 days. qPCR was performed to analyze the expression of osteogenic genes. (**c**) Western blotting assay was performed using the indicated antibodies. (**d**) ALP staining was performed to estimate ALP activity as an indicator of osteoblast differentiation after treatment with policosanol and Com C. (**e**) MC3T3-E1 cells were treated with policosanol and Com C for 21 days. The cells were stained with ARS solution to reveal extracellular mineralization. * *p* < 0.05, ** *p* < 0.01 compared with the untreated control; ^#^ *p* < 0.05, ^##^ *p* < 0.01 compared with the policosanol-treated group. Data represent the mean ± SD of three individual experiments.

We identified whether policosanol activates AMPK in osteoblast. As shown in Figure 2a, the addition of policosanol increased AMPK phosphorylation. Compound C (Com. C), an AMPK inhibitor, reduced the expression of policosanol-induced osteogenic genes and protein levels (Figure 2b,c). In addition, Com C was found to reduce policosanol-induced ALP activity and mineralization without osteogenic conditions (Figure 2d,e). These results indicate that policosanol induces osteogenic differentiation through AMPK activation.

### 3.3. INSIGs Mediated Policosanol-Induced Osteoblast Differentiation in MC3T3-E1 Cells

**Figure 3:**
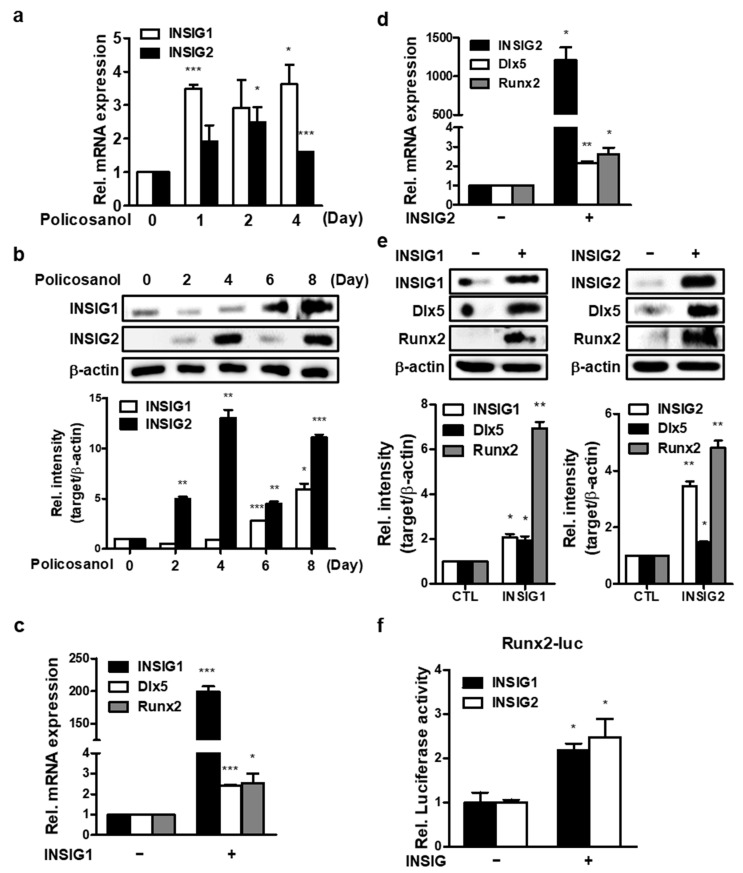
Policosanol-induced osteoblast differentiation is mediated by INSIG. (**a**) Cells were treated with 50 μg/mL policosanol for 4 days. qPCR was performed using total RNA isolated from MC3T3-E1 cells. (**b**) Western blotting assay was performed using total protein isolated from 50 μg/mL policosanol-treated cells. (**c**,**d**) Cells were transfected with the INSIG1 or -2 overexpression vector, and 48 h after transfection expression of mRNA encoding INSIG1 and -2 were measured by qPCR. (**e**) Western blotting assay was performed using total protein isolated from INSIG1 and -2 overexpression vector-transfected cells. (**f**) Cells were co-transfected with the INSIG1 or -2 overexpression vector, and Runx2-luc vector. At 48 h after transfection, Runx2 luc-activity was measured by luciferase assay. * *p* < 0.05, ** *p* < 0.01, and *** *p* < 0.001 compared with the untreated control. Data represent the mean ± SD of three individual experiments.

To investigate the effects of AMPK on INSIGs, INSIGs gene expression was evaluated after policosanol treatment in MC3T3-E1 cells. Policosanol induced the expression of *INSIG1* mRNA on the first and fourth days and induced the expression of the *INSIG2* mRNA on the second and fourth days. (Figure 3a). Moreover, policosanol also increased both INSIG1 and -2 expression at the protein level (Figure 3b). Then, to examine the effects of INSIGs on osteogenic differentiation, we transiently overexpressed INSIG1 and -2 in MC3T3-E1 cells. qPCR results showed that overexpressed INSIG1 and -2 increased osteogenic gene expression (Figure 3c), and Western blotting showed that levels of osteogenic marker proteins were also increased by INSIG1 and -2 overexpression (Figure 3d). In summary, the results showed that INSIGs act to increase osteogenic differentiation and that INSIGs expression is increased by policosanol.

### 3.4. Silencing of INSIGs Decreases Policosanol-Induced Osteogenic Differentiation

**Figure 4:**
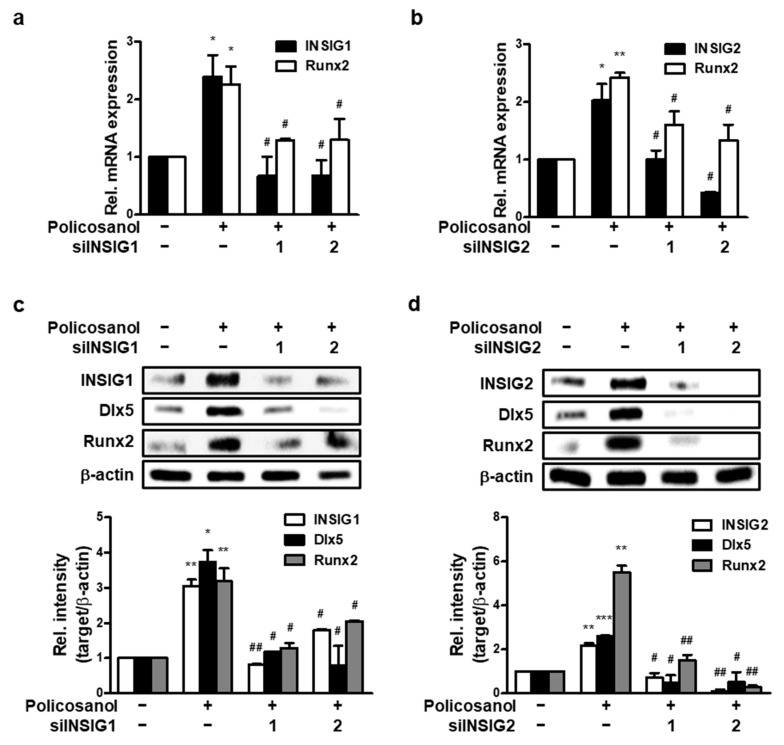
INSIG inhibition decreases policosanol-induced osteoblast differentiation. MC3T3-E1 cells were transfected with 100 nM INSIG1 or -2 siRNA. (**a**) *INSIG1* and *Runx2* mRNA expression were determined after transfection and then treatment with policosanol for 4 days. (**b**) mRNA expression of *INSIG2* and *Runx2* was measured using qPCR. (**c**,**d**) Total protein was extracted after the treatment of policosanol for 4 days. * *p* < 0.05, ** *p* < 0.01, *** *p* < 0.001 compared with the untreated control. ^#^ *p* < 0.05, ^##^ *p* < 0.01 compared with the policosanol-treated group. Data represent the mean ± SD of three individual experiments.

To further investigate the effect of INSIGs on osteoblast differentiation, we prepared two types of INSIG1 and two siRNAs and transfected them into MC3T3-E1 cells. qPCR was performed to measure the mRNA expression of each *INSIG* and the key regulator of osteoblast differentiation, *Runx2*. Gene expression was compared between untreated cells and those treated with policosanol. The knockdown of INSIG1 decreased policosanol-induced *INSIG1* and *Runx2* mRNA expression (Figure 4a). Similarly, the silencing of INSIG2 decreased the policosanol-induced increase in *INSIG2* and *Runx2* mRNA expression (Figure 4b). The siINSIGs also decreased policosanol-induced INSIG, Dlx5, and Runx2 expression at the protein level (Figure 4c,d). These findings suggest that INSIG knockdown downregulates Runx2 expression.

### 3.5. Policosanol-Induced INSIG Expression Is Dependent on Phosphorylation of AMPK

**Figure 5:**
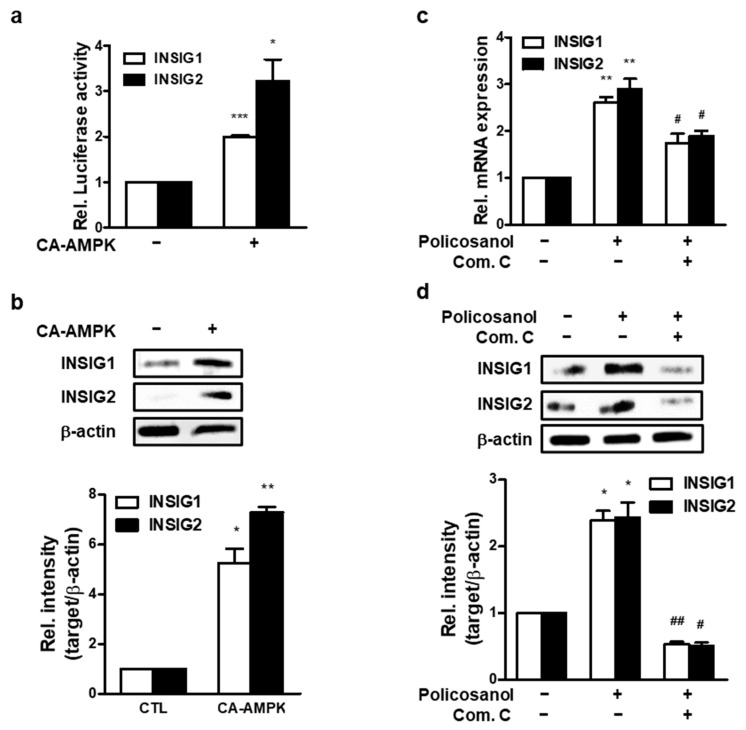
Policosanol induces AMPK-mediated INSIG expression. (**a**) MC3T3-E1 cells were transiently transfected with CA-AMPK for 48 h, after which mRNA expression was analyzed by qPCR. (**b**) After transfection with CA-AMPK for 48 h, cells were harvested to analyze the protein level by Western blotting using indicated antibodies. (**c**,**d**) MC3T3-E1 cells were treated with 50 μg/mL policosanol and/or 1 μM Com C. After treatment, mRNA expression was determined using qPCR, and the protein level was measured by Western blotting assay. * *p* < 0.05, ** *p* < 0.01, *** *p* < 0.001 compared with the untreated control; ^#^ *p* < 0.05, ^##^ *p* < 0.01 compared with the policosanol-treated group. Data represent the mean ± SD of three individual experiments.

To determine whether AMPK regulates INSIG, CA-AMPK was transfected into MC3T3-E1 cells. Activated AMPK increased mRNA and protein levels of INSIG1 and -2 (Figure 5a,b). Next, AMPK activity was inhibited using Com C. As a result, policosanol-induced *INSIGs* mRNA expression was decreased (Figure 5c), as was INSIG protein expression (Figure 5d). These data indicate that the expression of INSIGs induced by policosanol is mediated by phosphorylation of AMPK.

### 3.6. Policosanol Induces Zebrafish Fin Regeneration

**Figure 6:**
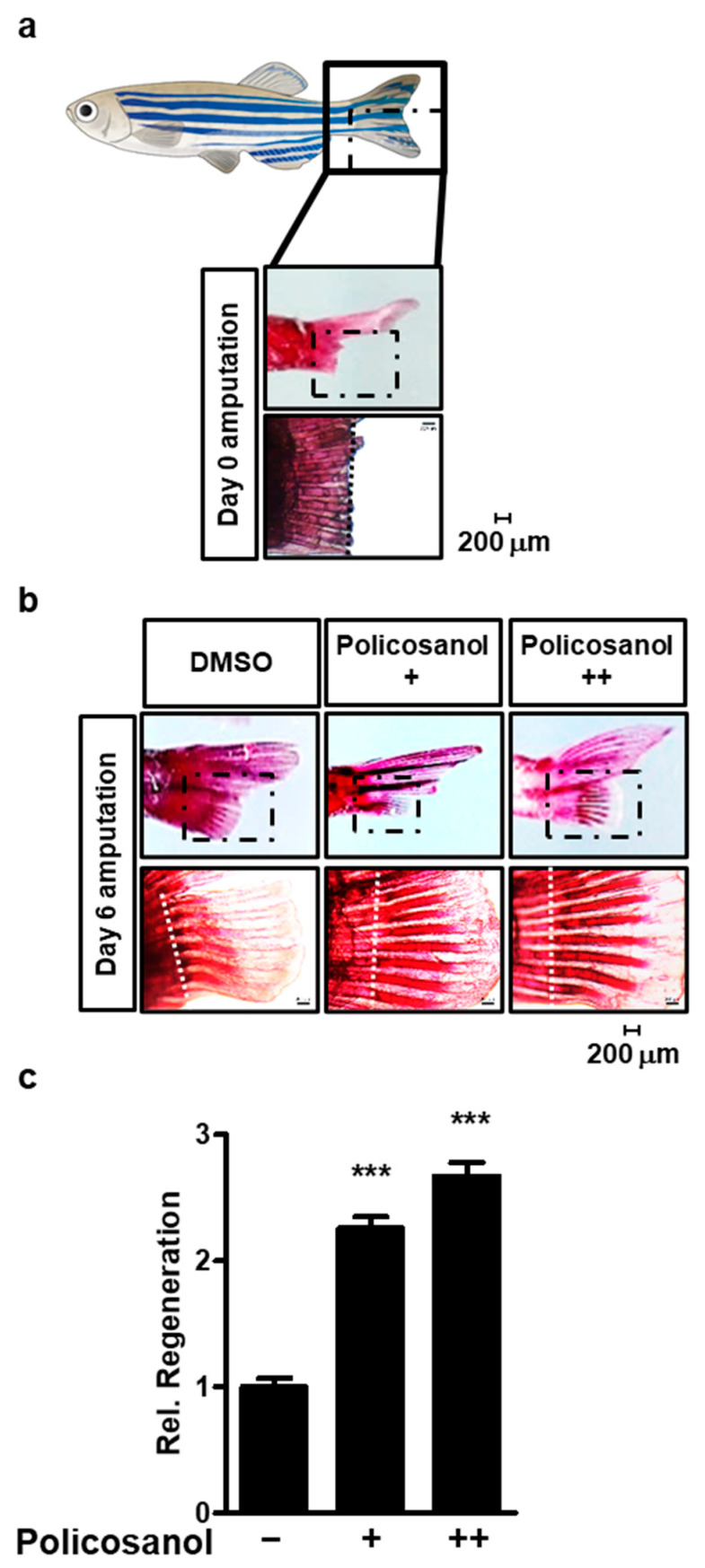
Policosanol induces regeneration of zebrafish fins. (**a**) Caudal fin rays of adult zebrafish that have been amputated at day 0. ARS staining to reveal mineralization of the caudal fin rays of adult zebrafish (top, total caudal fin rays; bottom, enlarged part). (**b**) Caudal fin rays of adult zebrafish were cut, and the fish were treated with either policosanol for 6 days (+: 50 μg/mL and ++: 100 μg/mL). (**c**) Caudal fin rays of adult zebrafish were quantified and compared between the control and policosanol-treated group. Scale bar, 200 μm. *** *p* < 0.001 compared with the control zebrafish. Data represent the mean ± SD of three individual experiments.

Our results established that policosanol positively regulates osteoblast differentiation. An experiment was conducted using a zebrafish model to confirm whether the effect of policosanol was shown in vivo. First, after cutting the tail fin of the zebrafish, it was confirmed that ARS staining was performed (Figure 6a). Next, zebrafish were treated with 50 μg/mL and 100 μg/mL of policosanol, and then zebrafish caudal fins were stained. As a result, both 50 μg/mL and 100 μg/mL policosanol increased zebrafish caudal fin regeneration (Figure 6b). As a result of quantifying the regenerated fins based on the cut surface, policosanol increases the regeneration of zebrafish caudal fins in a dose-dependent manner (Figure 6c).

## 4. Discussion

This study demonstrates the osteogenic effect of policosanol and suggests that AMPK-mediated INSIG expression is a mechanism by which policosanol increases osteoblast differentiation. Policosanol upregulated osteogenic gene expression and AMPK phosphorylation. Furthermore, modulation of AMPK activation altered osteoblast differentiation, and this change was indicated by the regulation of INSIGs expression.

Osteoblast differentiation proceeds with high expression of osteogenic genes such as Dlx5 and Runx2 [34,35]. Runx2 is known as a key regulator of osteoblast differentiation. In Runx2-deficient mice, bone formation is poor because the necessary late expression of genes, such as osteocalcin (OC) and bone sialoprotein (BSP), does not express [36]. Increased expression of these genes in osteoblasts indicates the induction of osteogenesis, and in the present study, we found that policosanol increased the expression of Dlx5 and Runx2, confirming that policosanol had a positive effect on osteoblast differentiation. 

We previously reported that policosanol inhibited calcification of vascular smooth muscle cells [32]. However, this study suggests that policosanol increases osteoblast differentiation. In general, mineralization means that calcium is deposited and hardened in hard tissues such as teeth and bones, and calcification refers to abnormal calcium deposition in soft tissues, resulting in a pathological phenotype [37]. In addition to this study, we have reported the results of studies in which other natural products increased bone mineralization but inhibited vascular smooth muscle cell calcification [38,39]. Thus, our findings suggest that policosanol acts by increasing mineralization in hard tissue bone and inhibiting calcification in soft tissue. 

There are many reports that AMPK activation increases osteoblast differentiation [33,40]. In addition to these findings, many studies show that natural chemical compounds stimulate osteoblast differentiation by inducing the activity of AMPK [8,41]. According to our previous study, activation of AMPK induces phosphorylation of Smad1/5/8, which plays an important role in the BMP signaling pathway, and increases the expression of Dlx5 gene, thereby increasing osteoblast differentiation [33]. Since Compound C (Com. C) is a well-known selective inhibitor of AMPK, blocking the activation of AMPK can inhibit osteoblast differentiation. Similarly, our results showed that the expression of osteogenic genes Dlx5 and Runx2, which were increased by policosanol, was decreased by Com. C. AMPK activation is also involved in the mechanism by which policosanol inhibits cholesterol synthesis [42,43]. Our data showed that policosanol induces AMPK activation in osteoblasts, which promoted osteoblast differentiation. CA-AMPK-phosphorylated AMPK stimulated osteogenic differentiation. These findings indicate that policosanol acts on osteoblast differentiation through activation of AMPK. 

There are various reports of the effects of policosanol. Among them, the most well-known effect is to inhibit cholesterol biosynthesis. High cholesterol levels reduce the proliferation and differentiation of osteoblasts, thereby inhibiting bone formation and increasing the risk of osteoporosis [44]. In addition, there are several reports that INSIG inhibits cholesterol biosynthesis [25,45]. Therefore, we hypothesized that INSIG may be involved in the mechanism of policosanol-induced osteoblast differentiation. Previous studies have reported that INSIG knockout mice maintain high cholesterol levels and have reduced bone formation due to accumulation of abnormal ciliary vesicles (CVs) [46]. In this study, the effect of INSIG on osteoblast differentiation through overexpression or knockdown was investigated, and overexpressed INSIG upregulated osteoblast differentiation markers. Moreover, knockdown of INSIG inhibited policosanol-induced osteoblast differentiation. The promoter activity of Runx2 was also increased by overexpression of INSIG, confirming that INSIG can increase the transcriptional activity of Runx2. However, additional research is needed to determine whether this phenomenon is a direct effect or whether it promotes the function of other transcription factors. This reveals that INSIG is involved in osteoblast differentiation by policosanol treatment. 

Regarding the relationship between AMPK and INSIG, it has been reported that activation of AMPK is involved in the post-transcriptional activity of INSIG [47]. It has also been shown that the NKB1-AMPK-INSIG signaling pathway has potential in treating non-alcoholic fatty liver disease (NAFLD) [48]. Interestingly, in this study, which is different from the results of previous studies, regulating AMPK activation regulated INSIG expression. Therefore, further studies are needed to find out how regulating the activation of AMPK can regulate the expression of INSIG.

The zebrafish body fin rays consist of osteoblast cells. If the caudal fin is amputated reconstruction requires new osteoblasts to be produced by osteoblast differentiation [49]. During the regeneration process, the fins of the zebrafish are dedifferentiated into osteoblastic progenitor cells, which serve as a source for bone restoration, and fin regeneration proceeds as the cells differentiate into osteoblasts [50,51]. Therefore, the increase in the degree of caudal fin regeneration in zebrafish can be seen as the active differentiation of osteoblasts. Our in vivo study demonstrated that treatment with policosanol regenerated amputated zebrafish fin rays, verifying its effect on osteoblast differentiation.

## 5. Conclusions

This study indicated the effect of policosanol on osteoblast differentiation and its molecular mechanism in vitro and showed the effect of policosanol using zebrafish in vivo. Policosanol upregulated expression osteogenic genes, such as Dlx5 and Runx2, via the expression of INSIGs through the activation of AMPK in the MC3T3-E1 preosteoblast cell line. In addition, it was found that regeneration of the amputated zebrafish caudal fin was also increased. Therefore, it is suggested that policosanol has a positive effect on osteoblast differentiation and bone formation. 

## Data Availability

The data that support the findings of this study are available from the corresponding author upon reasonable request.
